# Direct Measurement of Energy Dissipation in Nanoscale Tribomechanical Interfaces: Dissipative Transfer Steady State

**DOI:** 10.3390/ma19112258

**Published:** 2026-05-26

**Authors:** Dinh Dat Pham, Yuichi Otsuka, Yukio Miyashita

**Affiliations:** 1 Department of System Safety Engineering, Nagaoka University of Technology, 1603-1 Kamitomioka, Nagaoka 940-2188, Japan; dat19@vos.nagaokaut.ac.jp; 2 Department of Mechanical Engineering, Nagaoka University of Technology, 1603-1 Kamitomioka, Nagaoka 940-2188, Japan

**Keywords:** cyclic wear, scanning probe microscope, fretting wear, contact mechanics, adhesion

## Abstract

This study examines the development of a steady state in the cyclic wear process for various combinations of metallic and inorganic materials. Energy dissipation is widely acknowledged as a significant parameter in wear mechanisms. However, at the nanoscale, the linear correlation between energy dissipation and wear progression is not consistently applicable. In this study, experimental observations of cyclic wear between scanning probe microscopy (SPM) cantilevers and substrate displacement were conducted. Substrate vibrations were monitored using a laser Doppler vibrometer, which facilitated the direct estimation of energy dissipation at nanocontacts during cyclic loading. The wear rates of the substrates decreased with an increase in the number of cyclic loadings, indicating the formation of a transfer steady state at the interface. Symmetric contact mode, based on the viscoelastic behavior of the contact, and asymmetric mode, based on adhesion between the interfaces, are commonly observed. The asymmetric mode evolved in the later stages of cyclic wear, suggesting the transfer of the steady state between the interfaces. A linear relationship between energy dissipation and wear rates was still observed for metallic substrates, whereas a steady state was observed for inorganic materials. This difference can be attributed to material exchange at the interfaces.

## 1. Introduction

Reciprocating wear is a significant concern in load-bearing mechanical systems, affecting the performance, efficiency, and longevity of materials in diverse applications. Prior research has demonstrated that fretting wear can lead to substantial degradation of metallic materials and protective coatings employed in biomedical implants, thereby diminishing their long-term structural reliability and biocompatibility [1,2,3,4,5,6]. At smaller scales, wear processes have also been observed under intermittent contact conditions, underscoring the significance of transient interfacial interactions, even in non-continuous sliding systems [7]. Moreover, reciprocal wear has been shown to markedly affect the machining performance and tool degradation [8]. Understanding wear behavior is essential for ensuring operational reliability and reducing the risk of failure [9,10,11,12]. Numerous wear models have been formulated to predict the wear behavior under specific operational conditions in engineering systems [13,14,15,16]. These models generally establish empirical relationships between the wear rate and engineering parameters [17]. Nevertheless, a fundamental understanding of the underlying wear mechanisms remains indispensable. Macroscopic wear is often analyzed using Archard’s model, which suggests that material removal is proportional to the load and sliding distance [18,19,20]. This removal behavior can be characterized by an empirical coefficient dependent on the counterbody material and frictional conditions [21,22]. However, the predictive reliability is limited by the absence of explicit physical mechanisms. Atomistic wear models conceptualize material removal as a sequence of interfacial atomic reactions [23,24,25,26,27]. However, the role of deformation, particularly inelastic processes at the contact interface, has not been explicitly addressed. The significance of local deformations and interfacial modifications in nanoscale contacts has been highlighted [28,29,30,31]. Molecular dynamics studies have demonstrated that inelastic deformation and interfacial interactions can significantly alter the cyclic wear behavior [32]. Experiments conducted under high-pressure conditions have further emphasized the role of inelastic deformation, including amorphization and plastic flow, in governing interfacial processes [33,34,35,36]. Energy-based methodologies offer a promising framework for investigating the wear mechanisms. In fretting wear systems, dissipated energy has been extensively utilized as an indicator that connects frictional interactions to material removal behavior and the evolution of wear volume [15]. Beyond mere frictional dissipation, recent studies have proposed that accounting for the energy dissipated through inelastic deformation can enhance the prediction of wear resistance in strain-hardened materials and provide deeper insights into adhesive wear mechanisms [19,37]. Macroscopic fretting wear was observed, wherein the volume of the removed material scaled linearly with the cumulative energy dissipated over the loading cycles [16,38]. At the nanoscale, the Scanning Probe Microscope (SPM) technique facilitates the investigation of wear behavior [30,39]. D. Y. Li and Hongbo Pan have reported that wear behavior at the cantilever can proceed via atom-by-atom removal or plastic deformation, contingent upon the loading conditions, resulting in distinct energy dissipation characteristics [40]. Energy dissipation estimation using conventional SPM approaches is typically inferred from spatially and temporally averaged cantilever oscillation data [41,42]. However, averaging cannot differentiate the response arising from various interaction events, such as adhesion, intermittent contact, or bond rupture. Consequently, establishing a mechanistic link between energy dissipation and nanoscale wear is challenging. However, recent studies have demonstrated that laser Doppler vibrometry (LDV) enables the non-contact measurement of surface vibrations with nanometer resolution and high temporal sensitivity, rendering it suitable for probing nanoscale dynamic responses [43,44].

In this study, a measurement framework was established wherein the substrate velocity, obtained via LDV, and the cantilever oscillation were simultaneously acquired using a unified data acquisition system. The interfacial processes governing energy dissipation and their relationship with the wear rate under cyclic loading conditions for representative material pairs, including Si/Ti, Si/HAp, Ti/Ti, and Ti/HAp, were investigated. The incorporation of LDV provides real-time measurements of substrate motion, enabling direct probing of the coupled dynamics at the contact. We found that the interfacial processes were monitored through distinct contact modes identified based on the energy dissipation and phase difference between the cantilever and substrate vibrations. Heterogeneous cyclic wear was observed across different material pairs, underscoring the significant influence of material-dependent inelastic deformation and interfacial modification on the wear behavior. The relationship between the energy dissipation rate and wear rate was established, revealing a divergence in the wear mechanisms associated with the dissipative material transfer steady state at nanoscale contacts. These findings provide direct insights into the coupling between contact dynamics and material transfer, contributing to the development of physically grounded wear models that incorporate energy dissipation and interfacial evolution.

## 2. Materials and Methods

### 2.1. Measurement Apparatus

**Figure 1:**
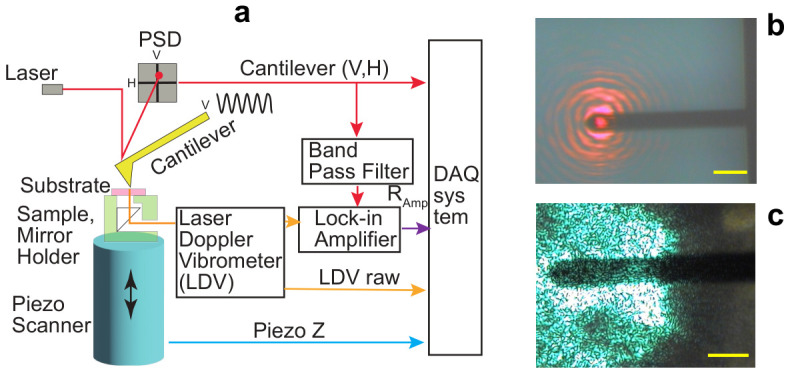
Experimental configuration of AFM-LDV Measurement System. (**a**) Schematic representation of the integrated AFM-LDV setup and data acquisition process. (**b**) Alignment of the cantilever on a designated region of the substrate. (**c**) Positioning of the LDV laser spot on the substrate surface. The yellow scale bars in (**b**) and (**c**) represent 10 mm, respectively.

The cyclic wear behavior at nanoscale contact was examined using a scanning probe microscopy system (SPM-9700, Shimadzu, Kyoto, Japan) as shown in Figure 1a. The deflection of the cantilever was monitored using a position-sensitive detector (PSD) of SPM system, which provided high-resolution data on the tip–sample interaction forces. To capture the dynamic deformation of the substrate surface, an external laser Doppler vibrometer (LDV) system (VibroFlex, Polytec GmbH, Waldbronn, Germany) was integrated with the SPM setup. The LDV signal represents the instantaneous effective velocity of the target region on the substrate surface, facilitating direct access to the dynamic response of the surface during cyclic loading. A band-pass filter was applied to the PSD signal to extract a clean and stable reference signal corresponding to the fundamental oscillation component of the cantilever. This filtered signal was subsequently employed as the reference input for a lock-in amplifier to ensure phase-sensitive detection with a reduced noise level. With phase-locking of the lock-in amplifier, the raw apparent surface velocity signal from the LDV and the signal that was synchronized by phase-locking were simultaneously recorded using a LabVIEW PXIe-based data acquisition system (PXIe-1083 controller and PXIe 5105 oscilloscope, National Instruments, Austin, TX, USA). Simultaneously, the adjustment of the piezo scanner was synchronized with the cantilever deflection, allowing for the investigation of the substrate surface topology.

### 2.2. Samples Preparation

Two distinct types of cantilevers were employed to investigate the material dependency of the wear behavior at the nanoscale. The first was a commercial silicon (Si) cantilever that served as a reference probe. The second cantilever was fabricated by coating a layer of pure titanium (Ti) onto a commercial Si cantilever using a sputtering deposition technique, which facilitated the examination of the Ti–Ti and Ti–HAp interactions.

A Ti-coated substrate was produced by depositing a pure Ti thin film onto a glass slide (thickness: 0.13–0.17 mm) by sputtering. Additionally, a hydroxyapatite (HAp) substrate was produced from HAP-100 powder (Taihei Chemical Co., Ltd., Osaka, Japan) using a spark-plasma sintering (SPS) system (LABOX-650F-N, Sinterland, Niigata, Japan). The HAp specimen was machined into a plate with dimensions of 2.0×2.0×0.5 mm^3^. The surface of the HAp substrate was polished to achieve a surface roughness of less than 0.05 μm, ensuring a minimal topographical influence on the wear behavior. To define the observation region, the surfaces of both types of substrates were marked using ultraviolet laser processing with a ProtoLaser U4 system (LPKF, Garbsen, Germany), which enabled precise delineation of the survey area for subsequent measurements.

### 2.3. Nano Wear Examination

Nanowear experiments were conducted using four tip/substrate combinations: Si/Ti, Si/HAp, Ti-coated Si/Ti, and Ti-coated Si/HAp substrates. Prior to measurement, the alignment of the LDV examination spot and the cantilever scanning region was achieved owing to the laser-marked patterns on the substrate surface, ensuring spatial consistency between the LDV measurement and contact interaction as shown in Figure 1b,c. A square area measuring 50 × 50 nm^2^ on the substrate surface was repeatedly scanned at a frequency of 1 Hz to induce and monitor the nanoscale cyclic wear. The cantilevers were operated near their fundamental resonance frequencies, approximately 12–13 kHz for the Si cantilever and within the range of 30–34 kHz for the Ti-coated Si cantilever. For each tip/substrate pair, four sequential scans were conducted to assess the progression of the wear rate as a function of the number of loading cycles. These repeated scans utilized the same tip and substrate pair and, therefore, did not constitute independent replicate experiments with replaced specimens. Data acquisition was synchronized using the trigger signal generated by the lock-in amplifier, ensuring that both the cantilever deflection and substrate velocity signals were recorded under phase-locked conditions.

### 2.4. Data Analysis

#### 2.4.1. Energy Dissipation Estimation

The approximation error of the elliptical fitting was quantitatively evaluated to assess the validity of phase-space representation. If the fitting error exceeded twice the standard deviation of the error distribution, the corresponding data segment was deemed to exhibit abrupt changes associated with intermittent or unstable contact conditions. Utilizing the extracted geometric parameters of the ellipses in conjunction with statistical measures of the fitting error, a clustering analysis was performed to discern distinctions in the contact regimes or morphological differences in the tip–substrate interaction. The obtained relationship between the cantilever vertical force $F_{cant}$ and the substrate velocity $v_{subs}$ was then analyzed.

The mechanism of energy dissipation was predominantly ascribed to a viscous force, represented by $F_{cant}=\gamma v_{cant}$, where γ signifies the effective damping coefficient and $v_{cant}$ denotes the cantilever velocity. The energy dissipated per cycle, $E_{cycle}$, which is proportional to the area of the elliptical trajectory in the force–velocity plane, was assessed using Equation (1).

(1) $$E_{cycle}=\int_{0}^{T}\gamma v_{cant}v_{subs}\;dt=\gamma \int_{0}^{T}v_{subs}\;dz_{cant}=\frac{\gamma }{k}\int_{0}^{T}v_{subs}\;dF_{cant}$$

Here, *T* denotes the vibration period, *k* denotes the effective stiffness of the cantilever, and $\int_{0}^{T}v_{subs}\;dF_{cant}$ corresponds to the area of the elliptical trajectory in the force–velocity plane. The representative energy dissipation per cycle, denoted as $E_{d}$, for each scan can be determined by calculating the mean of the areas of the elliptic curves across *N* detected cycles of the scan, as shown in Equation (2).

(2) $$E_{d}=\frac{\sum E_{cycle}/\frac{\gamma }{k}}{N}$$

#### 2.4.2. Wear Rate Evaluation

The variation in surface height between successive scans was assessed using topographical images processed with the Gwyddion 2.70 software [45]. To ensure accurate comparisons, the background tilt of the raw topography images was corrected by applying second-order polynomial leveling with independent degrees in both the horizontal and vertical directions of the images. A differential height map for the *i*-th scan was generated by subtracting the height data of the 1st scan from those of the *i*-th scan. The average wear depth for the *i*-th scan, denoted as $D_{i}$, was determined by calculating the mean height difference Δz across $N_{pixel}$ pixels within 11 parallel strips on the height map. Each strip had a width of 1 pixel, was separated by 10 nm, and was oriented perpendicular to the scanning direction of the electron beam. The average wear depth was computed using Equation (3).

(3) $$D_{i}=\frac{\sum \Delta z}{N_{pixel}}$$

Subsequently, the conventional wear rate $W_{con}$ for a scan was derived from the difference in the average wear depth between two consecutive scans, as shown in Equation (4).

(4) $$W_{con}=\frac{(W_{i}-W_{i-1})A}{N_{c}\pi r_{c}^{2}}$$

If considering wear as material exchange between both 2 objects, an absolute wear rate $W_{abs}$ should be estimated using Equation (5):

(5) $$W_{abs}=\frac{|W_{i}-W_{i-1}|A}{N_{c}\pi r_{c}^{2}}$$

where A represents the scanned area, quantified as $2500\;{\mathrm{nm}}^{2}$, $N_{c}$ denotes the number of cycles per scan, specified as $1.7\times 10^{5}$ cycles. In the current analysis, contact radius $r_{c}$ was approximated as a unit length and employed as a normalization factor to facilitate the comparison of wear evolution across different scanning conditions by correlating the scanned area with the accumulated apparent contact area during the repeated cycles. The actual contact radius was not directly measured, and no static contact mechanics model was applied because the true contact geometry was anticipated to evolve dynamically during oscillatory loading owing to the coupled effects of tip wear, tip blunting, adhesion, and material transfer. Consequently, the approximation of $r_{c}$ introduces systematic uncertainty into the quantitative estimation of the wear rate, particularly because progressive tip blunting may increase the true contact area during repeated scans. Therefore, the calculated wear rate should be interpreted as an effective normalized quantity, rather than an absolute local wear parameter.

### 2.5. Characterization of Wear Trace

The regions designated as contact zones, specifically the tips of the cantilevers and laser-marked areas on the substrate surface, were examined following the nanowear experiments. Owing to the limitations associated with electrical conductivity, the morphology of the substrate surface was examined using interferometric and optical microscopy. In addition, the morphology of the cantilever tips was characterized using scanning electron microscopy (SEM) to assess wear-induced modifications.

## 3. Results

### 3.1. Validation of Measuring Energy Dissipation

**Figure 2:**
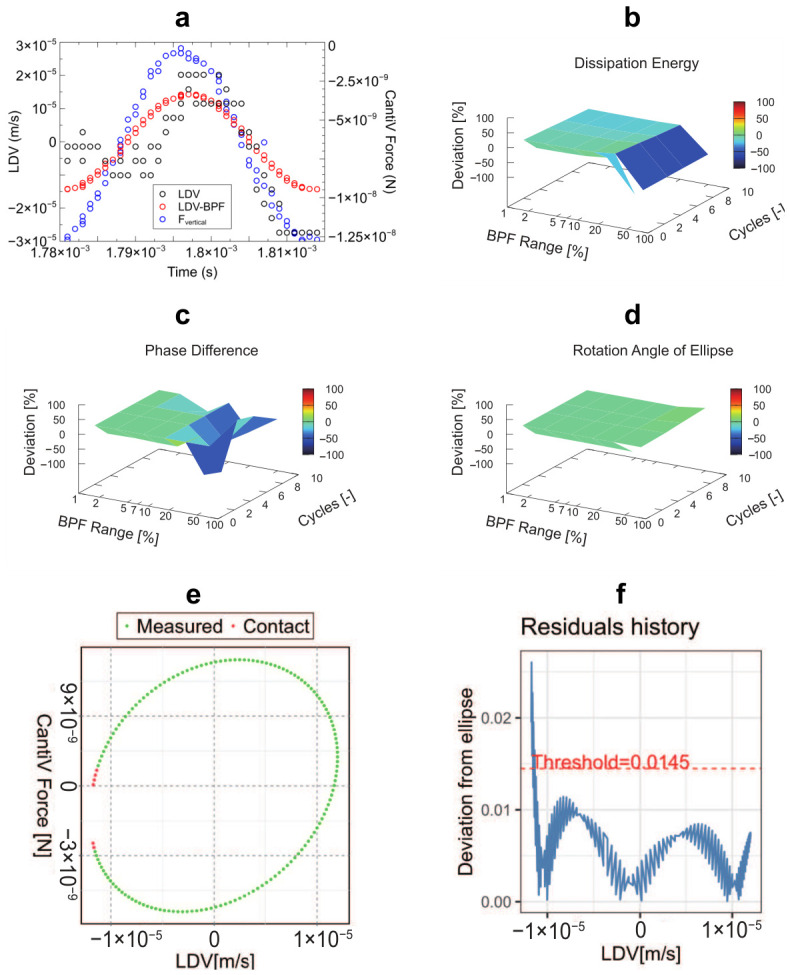
Estimation of energy dissipation and sensitivity of signal-processing parameters. (**a**) Band-pass filtering of the LDV signal for sinusoidal reconstruction. (**b**–**d**) Influence of the band-pass filter (BPF) frequency range and number of averaging cycles on the fitted Lissajous ellipse parameters, including (**b**) enclosed area, (**c**) phase difference, and (**d**) slope angle. The parameter values are presented relative to the single-cycle result obtained with a 10% BPF range, which was used as the reference condition. (**e**) Representative velocity–force Lissajous curve obtained from the Si/Ti interface. (**f**) Corresponding residual evolution used for contact-state estimation of the fitted curve.

The waveform of the substrate surface velocity was synchronized with the cantilever response through bandpass filtering (BPF) (Figure 2a). Although the LDV signal was acquired under phase-locked conditions, nonharmonic components were observed, which introduced deviations from the ideal sinusoidal motion, distorting the phase relationship between the substrate velocity and cantilever excitation if not properly filtered. After filtering, the reconstructed velocity signal (LDV-BPF) satisfies the assumption that the substrate oscillates at the same fundamental frequency as the excitation induced by the vertical force of the cantilever $F_{vertical}$.

The stability and sensitivity of the signal-processing procedure were assessed by analyzing the effects of the BPF bandwidth and the number of averaging cycles on the fitted Lissajous ellipse parameters (Figure 2b–d). A single cycle with a 10% BPF range was used as the baseline, providing a consistent reference despite phase variability. Increasing the number of averaging cycles resulted in a reduced estimated area owing to the heightened sensitivity to smaller internal loops within the Lissajous trajectory. Therefore, a single-cycle evaluation was deemed sufficient to capture the maximum energy dissipation value. The phase difference between the velocity and force waveforms (Figure 2c) demonstrated sensitivity to the signal processing conditions, particularly the bandwidth of the BPF and the number of averaging cycles. Depending on the angular convention employed to represent the fitted ellipse, the phase definition may appear inverted. Despite this variability, the estimated phase difference became relatively stable when the BPF bandwidth was restricted to 10% of the fundamental frequency, thereby supporting the filtering range adopted in this study. Averaging over multiple cycles may enhance the robustness of the analysis; however, in this study, a single-cycle evaluation was adopted to preserve transient dissipative features and capture the maximum apparent energy dissipation associated with individual loading events. The slope angle of the fitted ellipse (Figure 2d) remained nearly constant with respect to both the BPF bandwidth and the number of averaging cycles. Given that the slope angle reflects the phase relationship between the substrate surface vibration velocity and cantilever displacement, this relatively stable behavior indicates that the overall phase-space orientation of the contact response is less sensitive to the signal-processing conditions. Consequently, it may serve as a more robust descriptor of the interfacial dynamics.

The energy dissipated per cycle ($E_{cycle}$) was estimated from the velocity–force Lissajous curve corresponding to each loading cycle (Figure 2e). Prior to ellipse fitting, the signals were normalized by shifting the mean to zero and scaling the standard deviation to one. This normalization preserves the geometric shape of the trajectory while eliminating the bias introduced by offset drift and amplitude mismatch between the signals. Consequently, the subsequent elliptical approximation became stable, enabling the consistent extraction of the geometric parameters across cycles. The resulting loop evolved counterclockwise from the leftmost point, and the closure of the loop was ensured based on the phase of the LDV signal, with two points corresponding to the velocity sign reversal in each cycle.

The contact state was further evaluated based on the deviation between the measured LDV data and the fitted ellipse (Figure 2f). Contact instability was identified when the residual exceeded a threshold defined as twice the standard deviation of the residuals. Following the conventional sign convention for LDV measurements, a negative velocity corresponds to motion toward the substrate. The residual distribution in the representative cycle indicates that instability predominantly occurs at the beginning of the cycle, where the cantilever approaches the surface. Under ambient conditions, the cantilever is continuously influenced by capillary forces, resulting in an overall attractive interaction throughout the cycle (Figure 2a). Therefore, the detected instability marks the onset of effective mechanical contact, during which the applied load is sufficiently transmitted to the substrate to induce the material deformation and wear.

### 3.2. Evolution of Energy Dissipation During Cyclic Wear

**Figure 3:**
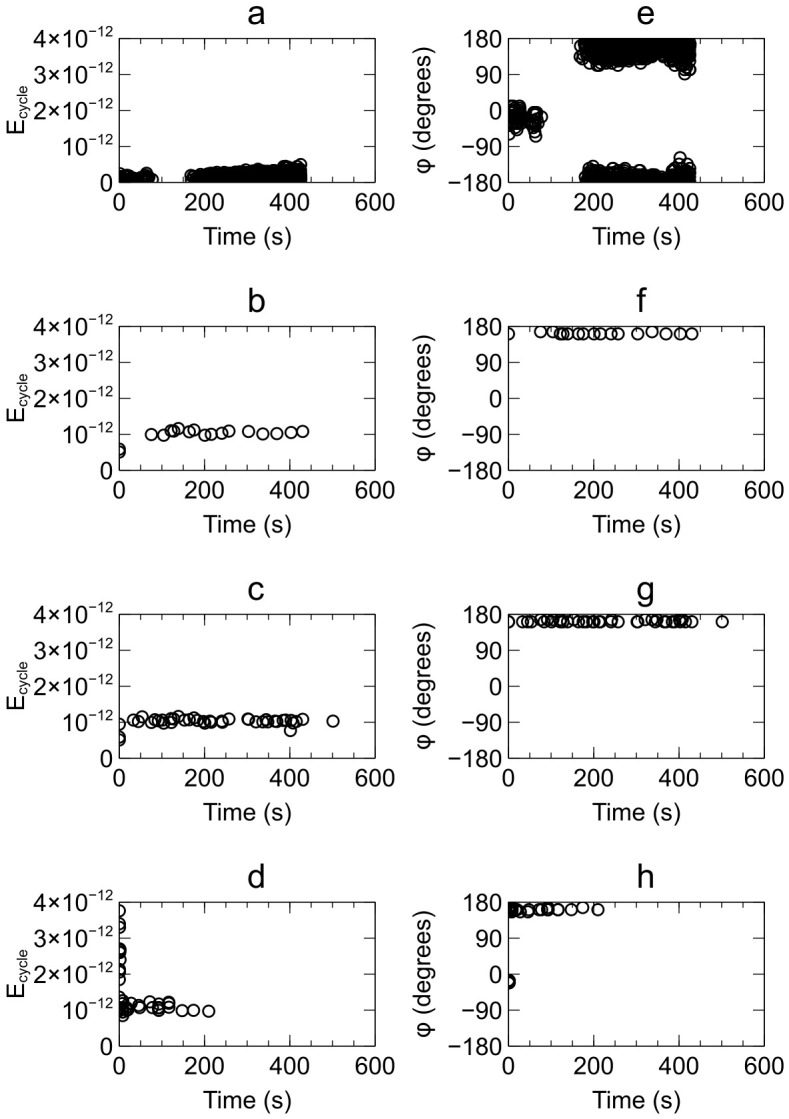
Evolution of dissipative behavior during Si cantilever scanning on the Ti-coated substrate. (**a**–**d**) Energ dissipation $E_{cycle}$ from scan 1 to 4. (**e**–**h**) Phase difference ϕ between $v_{subs}$ and $z_{cant}$ from scan 1 to 4.

**Figure 4:**
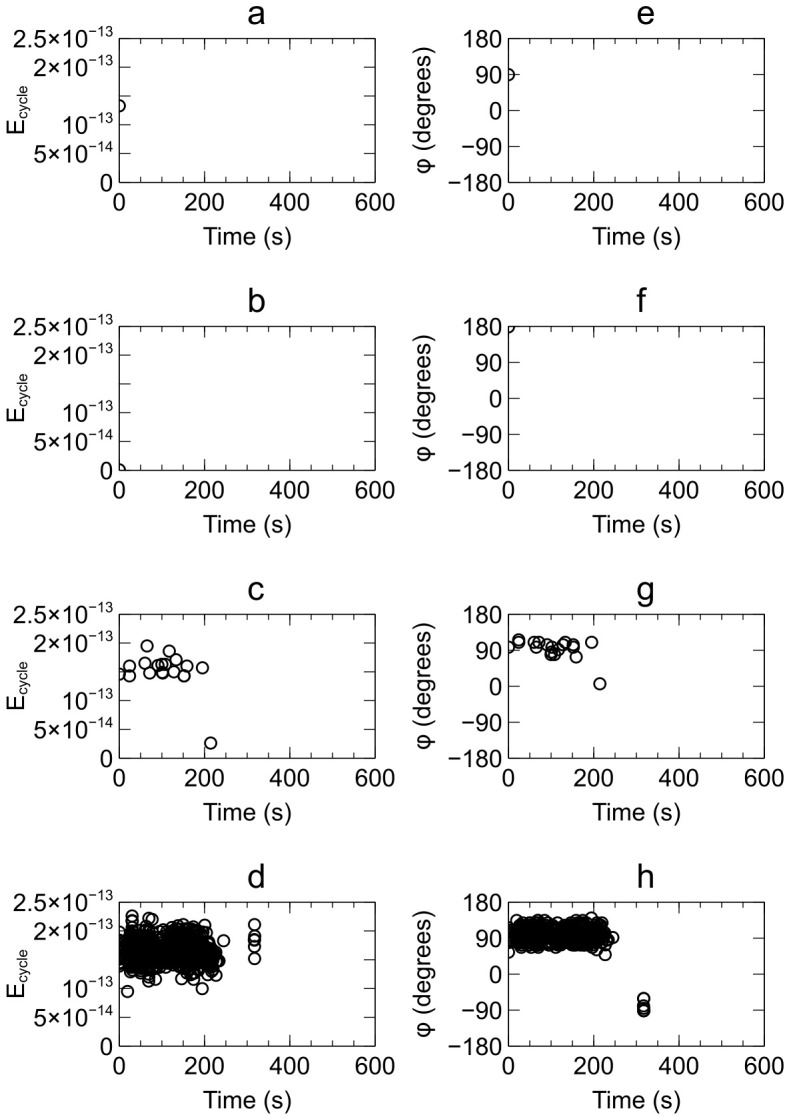
Evolution of dissipative behavior during Si cantilever scanning on the HAp substrate. (**a**–**d**) Energ dissipation $E_{cycle}$ from scan 1 to 4. (**e**–**h**) Phase difference ϕ between $v_{subs}$ and $z_{cant}$ from scan 1 to 4.

**Figure 5:**
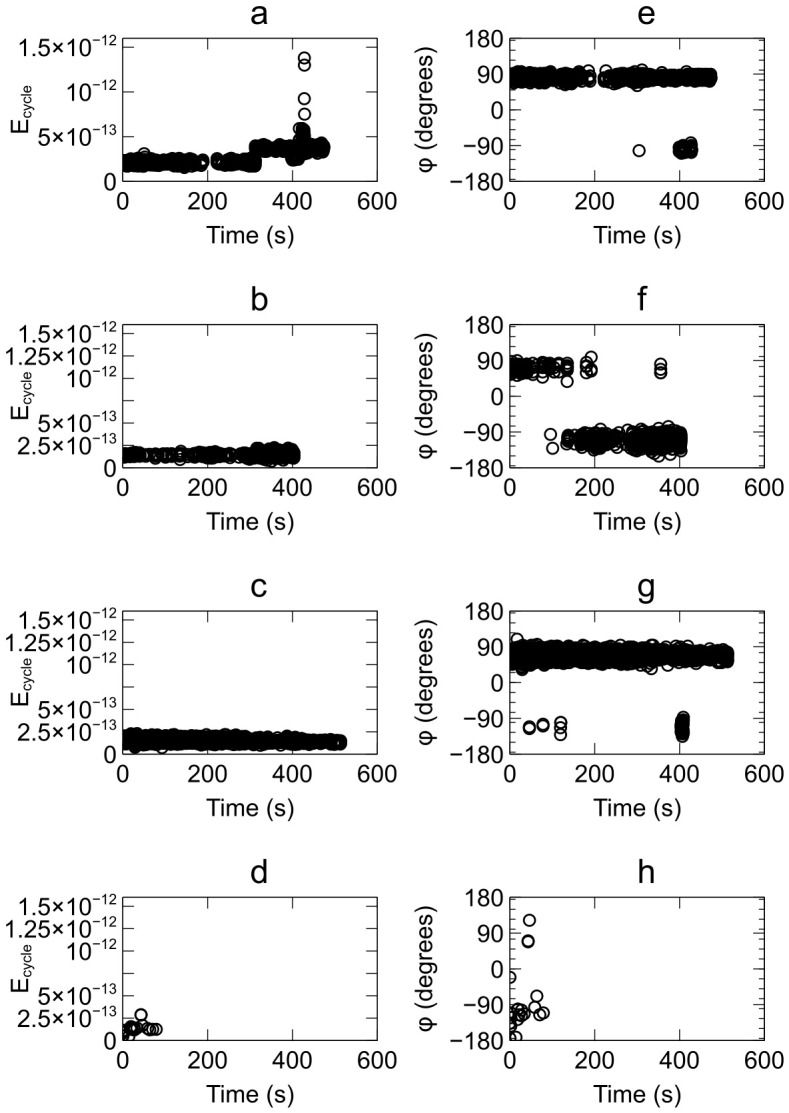
Evolution of dissipative behavior during Ti-coated Si cantilever scanning on the Ti-coated substrate. (**a**–**d**) Energ dissipation $E_{cycle}$ from scan 1 to 4. (**e**–**h**) Phase difference ϕ between $v_{subs}$ and $z_{cant}$ from scan 1 to 4.

**Figure 6:**
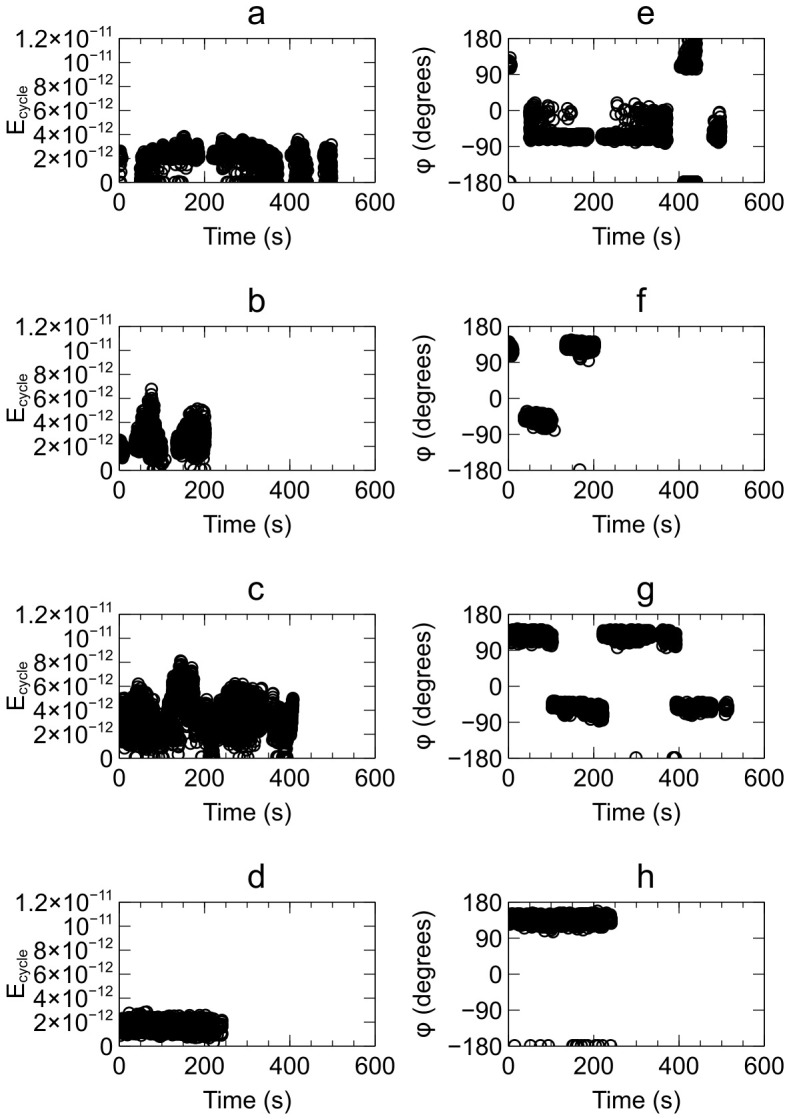
Evolution of dissipative behavior during Ti-coated Si cantilever scanning on the HAp substrate. (**a**–**d**) Energ dissipation $E_{cycle}$ from scan 1 to 4. (**e**–**h**) Phase difference ϕ between $v_{subs}$ and $z_{cant}$ from scan 1 to 4.

By analyzing the velocity–force curve, the progression of energy dissipation, denoted as $E_{cycle}$, at the substrate can be estimated across multiple scans (Figure 3, Figure 4, Figure 5 and Figure 6). The findings indicate the presence of distinct contact modes that can be qualitatively interpreted based on the phase relationship between the cantilever displacement and substrate surface vibration velocity. A phase lead of approximately π/2 between the substrate velocity and cantilever displacement may suggest a synchronized interfacial motion facilitated by adhesive coupling between the two surfaces, referred to here as an asymmetric contact mode. Conversely, phase differences that significantly deviate from this condition, particularly those exhibiting delayed-phase responses, align more closely with damped substrate oscillations associated with viscoelastic energy dissipation, which is termed a symmetric contact mode. Within each scan, both the dissipated energy and phase difference tended to cluster around characteristic values corresponding to specific contact states, indicating that energy dissipation reflects transitions in interfacial behavior beyond the average interaction force typically considered in conventional tribological measurements.

Figure 3 illustrates the progression of the dissipated energy and phase difference at the Si/Ti interface. The relatively stable dissipated energy observed throughout the repeated scans suggests that the predominant damage mechanism remained largely unchanged during the experiment. Correspondingly, the contact behavior is primarily associated with the symmetric mode, indicating that viscoelastic damping predominates the interfacial response. The velocity–displacement phase difference approaching π suggests that the vibrational response of the substrate occurs largely in opposition to the displacement direction of the cantilever, which may reflect a delayed elastic recovery or phase inversion associated with intermittent contact and damping-dominated oscillatory behavior.

Figure 4 reveals that the Si/HAp interface exhibits substantial variation in the dissipated energy, indicating the coexistence of multiple interaction sites of HAp. Notably, the asymmetric contact mode appears to dominate, despite the relatively weak adhesive tendency expected for Si and the brittle characteristics of HAp.

Figure 5 indicates that the Ti/Ti interface maintained a comparatively stable dissipated energy level, suggesting that the dominant damage mechanism remained relatively consistent throughout the experiment. However, the contact state frequently transitioned between asymmetric and symmetric modes, indicating repeated switching between adhesive and non-adhesive interaction states. This behavior reflects the intermittent nature of the contact during oscillatory sliding and underscores the importance of monitoring cycle-resolved contact states rather than relying solely on averaged interaction forces.

Figure 6 demonstrates the most complex behavior among the investigated material combinations. Significant fluctuations in the dissipated energy across repeated scans suggest that multiple inelastic damage mechanisms coexist and evolve dynamically during sliding. Simultaneously, the divergence of the observed contact modes may indicate the instability of the interfacial transfer layer formed during repeated contact. The combined evolution of dissipative behavior and phase-state variability suggests that the Ti/HAp interface undergoes continuously changing interfacial conditions governed by coupled wear, adhesion, and transfer layer processes.

**Figure 7:**
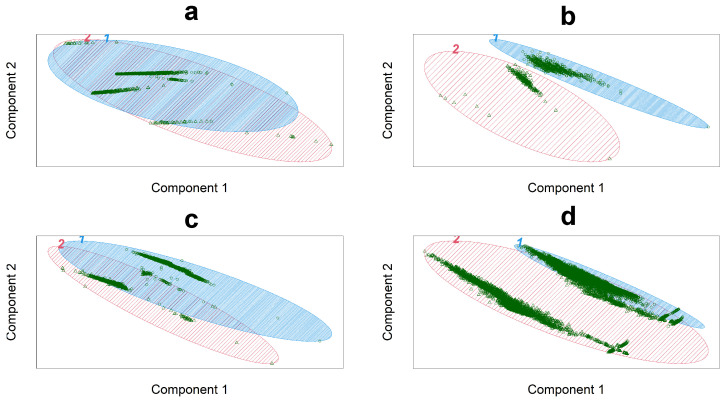
Clustering outcomes for various tip–substrate combinations. (**a**) Si/Ti, (**b**) Si/HAp, (**c**) Ti-coated Si/Ti, and (**d**) Ti-coated Si/HAp. Cluster 1 corresponds to the asymmetric mode, whereas Cluster 2 represents the symmetric mode.

Clustering analysis using the k-means method was applied for the dataset of 4 cycles (Figure 7). The optimal number of clusters was two, based on the Bayesian Information Criterion. The feature importance evaluated by a random forest model indicated that the phase difference and slope angle were the dominant variables governing the classification. Two clusters were determined, indicating two distinct contact modes.

**Figure 8:**
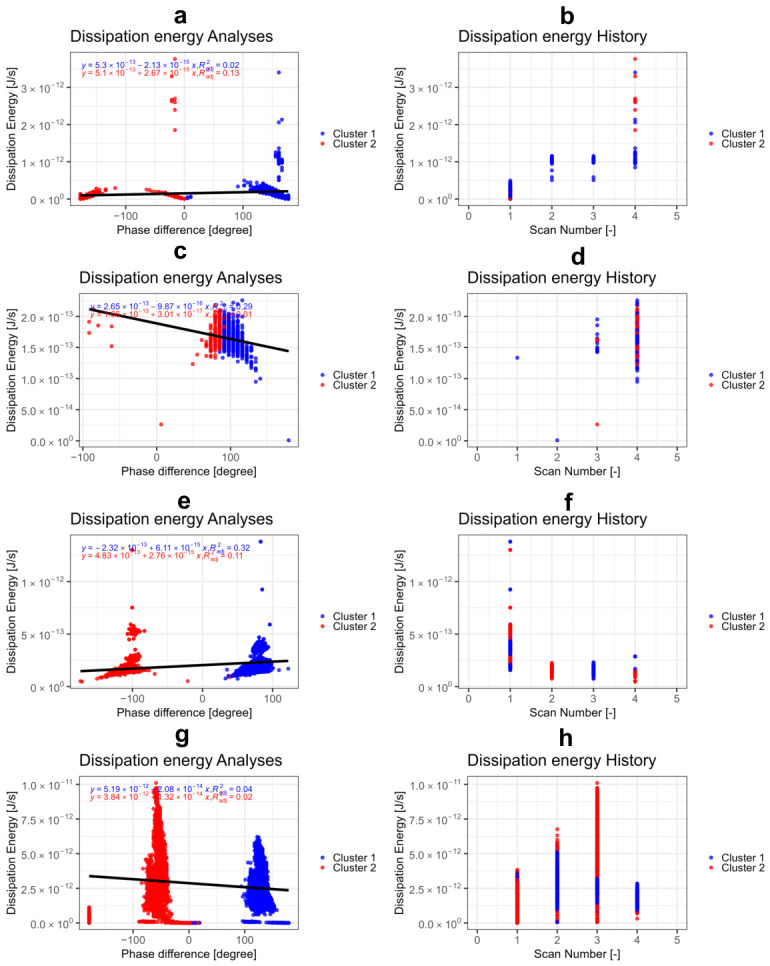
Characteristics of contact modes for different material pairs. (**a**,**c**,**e**,**g**) Distribution of energy dissipation as a function of phase difference, highlighting distinct contact modes. (**b**,**d**,**f**,**h**) Evolution of energy dissipation per scan cycle, indicating cycle-dependent behavior. The tip/substrate pairs, in order, are Si/Ti, Si/HAp, Ti-coated Si/Ti, and Ti-coated Si/HAp.

The clustering phenomenon is predominantly influenced by the sign of the phase difference (Figure 8a,c,e,g). Overall, the energy levels detected by the scans for most of the material pairs did not exhibit significant variations. The energy spike observed in the final scan of the Si/Ti case (Figure 8b) is likely attributable to the transfer of Ti clusters to the Si tip. In the Si/HAp case, the predominance of the adhesive mode suggests stable interfacial bonding, as evidenced by the subsequent scan (Figure 8d). The marked reduction in energy dissipation following the initial scan (Figure 4f) may be associated with the elastic shake-down of Ti. For the Ti-coated Si/HAp (Figure 8h), the non-monotonic behavior reflects the complexity of the contact nature, which is governed by the interplay between deformation and interfacial bonding.

### 3.3. Nano Wear Behavior

**Figure 9:**
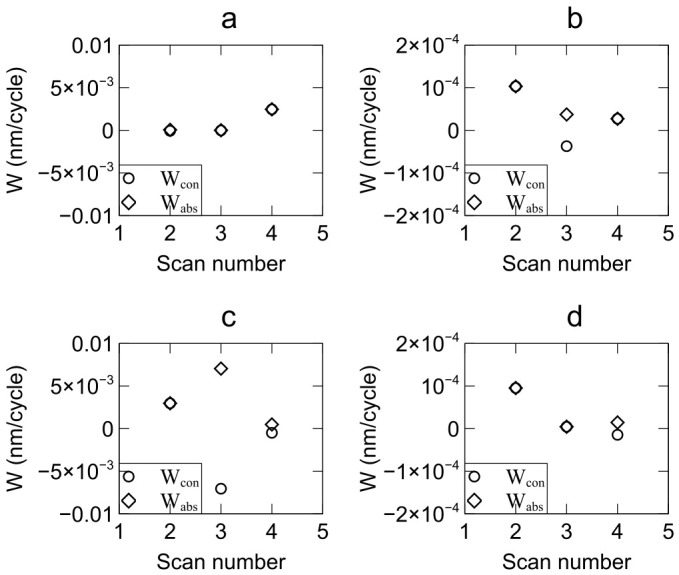
Heterogeneous cyclic wear behavior by scan of substrate in several tip/substrate pairs. (**a**,**c**) Ti substrates with Si and Ti-coated Si cantilever, respectively. (**b**,**d**) HAp substrates after test with Si and Ti-coated Si cantilever, respectively.

**Figure 10:**
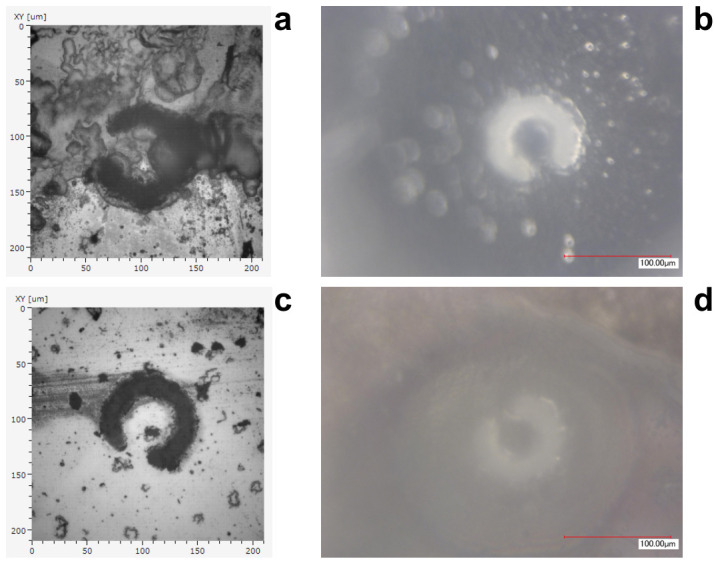
Observation of contact area of substrates after wear experiment. (**a**,**c**) Interferometric microscope images of Ti substrates after testing with Si- and Ti-coated Si cantilever, respectively. (**b**,**d**) Optical microscope images of HAp substrates after test with Si and Ti-coated Si cantilever, respectively.

The heterogeneous nanoscale wear behavior across repeated scans (Figure 9), underscores the instability of wear prediction based on empirical models. The conventional wear rate, denoted as $W_{con}$ and defined from the perspective of the substrate, exhibited negative values in several instances. This suggests an apparent net material transfer from the cantilever to the substrate. In this study, such material transfer was inferred from the measured increase in the substrate surface height following the scan. However, the direct characterization of interfacial material exchange processes, including potential diffusion and transfer-layer evolution, is beyond the resolution capabilities of the current experimental setup. Future investigations will require higher resolution analyses to address these aspects. Consequently, the absolute wear rate, $W_{abs}$, was considered to account for the total apparent material exchange rate, irrespective of the direction of transfer. Furthermore, the magnitude of wear per cycle being smaller than a single atomic volume suggests that wear events may be governed by the probability of bond rupture and atomic displacement. In this context, the contact state analysis introduced in Section 3.1 provides a promising framework for identifying an effective wear cycle. Additionally, the extremely low wear rates were consistent with the SEM observations of the substrate surfaces (Figure 10), where no morphological damage was observed on the surface. This agreement indicates that wear primarily occurs beyond the resolution of SEM.

**Figure 11:**
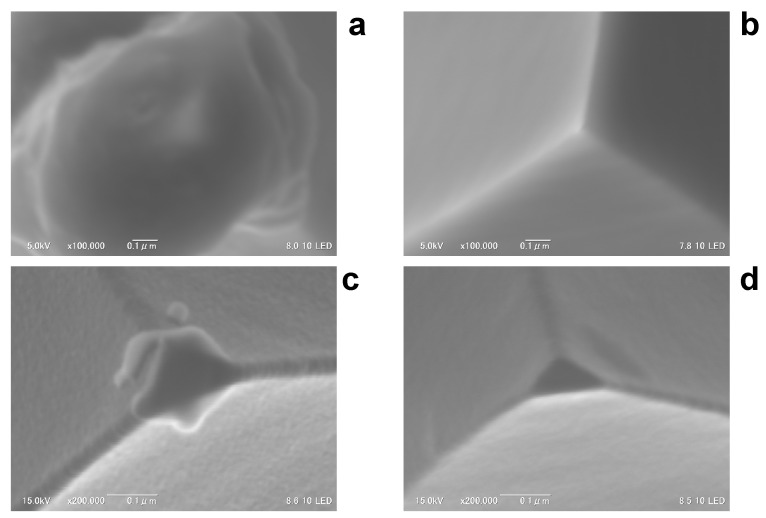
SEM observation of contact area of tips after wear experiment. (**a**,**b**) Si tips after test with Ti and HAp substrate, respectively. (**c**,**d**) Ti-coated Si tip after test with Ti and HAp substrate, respectively.

A pronounced difference in wear behavior is observed depending on the material of the contacting objects. The material observed on the cantilever tip after experiments on Ti substrates (Figure 11a,c) is likely Ti, suggesting strong adhesive interactions. This transferred material may subsequently be re-deposited onto the substrate, as indicated in scan 3 of Figure 9c. In contrast, the wear rate on the HAp substrates decreased with increasing scan number (Figure 9b,d), suggesting progressive stabilization of the contact interface. Although material transfer may still occur, either on the cantilever or the substrate, it remains below the resolution of the employed observation (Figure 10b,d and Figure 11b,d).

**Figure 12:**
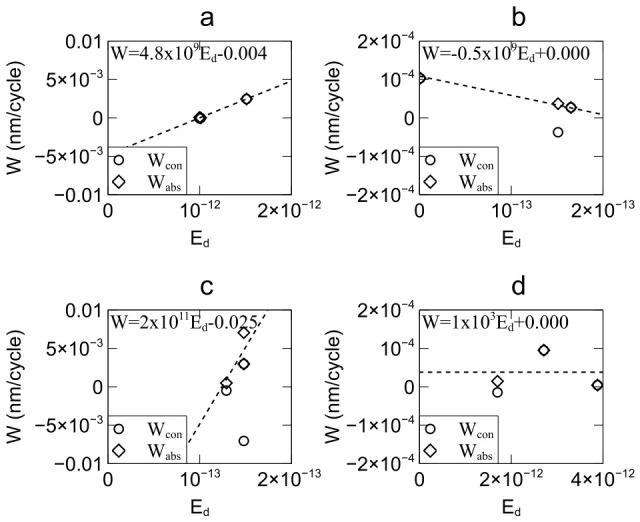
Dissipative wear behavior in several tip/substrate pairs. (**a**) Si/Ti, (**b**) Si/HAp, (**c**) Ti-coated Si/Ti and (**d**) Ti-coated Si/HAp.

The relationship between the energy dissipation rate and the wear rate (Figure 12), necessitates the formulation of an energy-approach wear model to account for heterogeneous behavior. The absence of a correlation between the dissipated energy ($E_{d}$) and conventional wear rate ($W_{con}$) indicates that a unilateral description of wear is inadequate to fully capture the physics of the system. In most instances, except for Ti-coated Si/HAp, a correlation between the absolute wear rate $W_{abs}$ and $E_{d}$ was observed, which aligns with the predictions of the energy-approach wear model (Figure 12a–c). The variations in the energy coefficients (the slope of the dissipated energy rate-wear rate curve) across different cases reflect the corresponding changes in the underlying mechanisms. For the Ti-coated Si/HAp pair, the divergence between the wear rate and energy dissipation trends suggests that the material transfer cannot be adequately described by a single mechanism.

## 4. Discussion

The findings facilitate a direct examination of wear behavior and energy dissipation under cyclic normal loading at the nanoscale. The energy dissipation and contact characteristics were reliably estimated using the BPF. Distinct contact modes were identified through clustering analysis, indicating that the interfacial interactions can be systematically classified. These interactions are governed by viscoelastic deformation and adhesion and should be characterized by phase differences. The effective wear rate varied with the scan cycle and material pair, highlighting the significant influence of interfacial interactions. Microscopy observations and estimations consistently demonstrated that cyclic nanoscale wear occurred at the atomic scale on both the tip and substrate. The correlation between the wear and energy dissipation rates suggests different wear mechanisms. Notably, the deviation of the Ti-coated Si/HAp case from the predictions of the conventional wear model indicates the complexity of interface modification in the nanoscale cyclic wear behavior.

### 4.1. Dissipative Transfer Steady State

**Figure 13:**
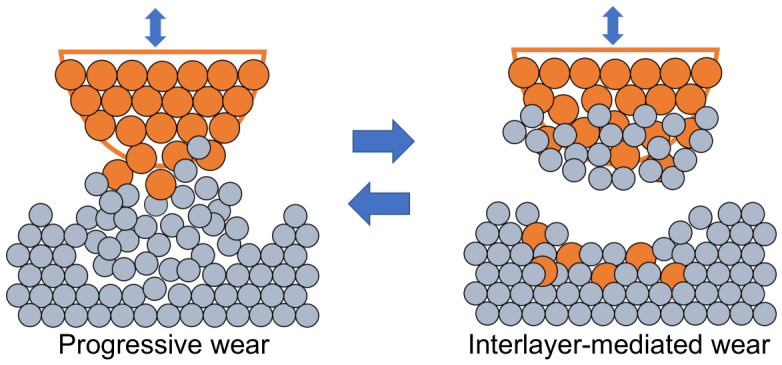
Schematic illustration of dissipative material transfer mechanisms at nanoscale contacts.

The mechanism of dissipative material transfer at steady-state nanoscale contacts can be interpreted as a dynamic balance between progressive wear and interlayer-mediated wear (Figure 13). In this framework, diffusion-driven wear is effectively regulated by the presence of an interlayer that accommodates deformation and reduces the interfacial driving forces. However, catastrophic wear can re-emerge when the loading conditions locally disrupt or remove the interlayer, exposing fresh surfaces and reactivating interaction-dominated material removal.

The balance of regimes is evidenced by the evolution of wear rate across scanning times. The stable behavior observed in the Si/HAp system, both in terms of the contact mode energy and wear rate, indicates the persistence of the interlayer-mediated regime. The low-energy wear coefficient further suggests an atom-by-atom wear manner, which can be attributed to the chemical inertness and high stiffness of the Si cantilever. In contrast, the anomalous adhesive wear observed in certain scans involving Ti substrates is likely associated with the higher ductility of titanium. Significant increases in the wear rate in these cases can be interpreted as interlayer delamination events, marking progressive wear. Meanwhile, the gradual stabilization observed in the Ti-coated Si/HAp system indicates an increasing contribution of interlayer-mediated wear, consistent with the trends reported in a prior simulation [32].

Notably, explaining the emergence of the steady-state behavior requires extending conventional energy-based wear models. In limiting cases, such as the dominance of interlayer-mediated wear, like in the Si/HAp case, or progressive wear, like in the Si/Ti and Ti-coated Si/Ti cases, this extension primarily involves redefining wear as a bidirectional process rather than unilateral material removal. In more complex systems, such as Ti-coated Si/HAp, where multiple wear regimes coexist and interact, a singular representative mechanism is inadequate. The apparent energy coefficient may vary according to the relative contributions of the different wear processes during each loading cycle. The mechanism proposed in this study suggests that certain cycles may involve severe wear events associated with interlayer detachment or rupture, leading to a relatively high energy dissipation per unit of wear. Conversely, other cycles may correspond to contact states temporarily stabilized by the transferred interlayer, where sliding occurs with reduced material removal and lower apparent dissipation efficiencies. Given that a single scan comprises numerous dynamic loading cycles with continuously evolving contact states, the coexistence and temporal alternation of these regimes can result in deviations from the linear relationship between the $E_{d}$ and $W_{abs}$. In such cases, resolving the specific contributions of distinct contact modes and their associated energy dissipation is essential for accurately describing and predicting wear behavior.

### 4.2. Implications of Dissipated Energy for Interfacial State Characterization

Direct observation of the actual contact state during dynamic loading remains a significant challenge; thus, the estimated dissipated energy may serve as a potential indicator of the evolving interfacial condition. The “real” contact area is anticipated to substantially influence the local stress distribution and associated energy dissipation mechanisms during repeated sliding. Tip blunting is commonly associated with a transition from highly localized plastic deformation to more gradual wear processes with redistributed dissipation pathways. In the present study, the material transfer observed on the cantilever tip may further complicate the estimation of the local deformation behavior by continuously changing the contact geometry. Theoretically, the contact area can be approximated from the cantilever loading conditions using non-adhesive or adhesive contact models; however, under dynamic oscillatory loading, the actual contact area is likely to evolve continuously owing to coupled wear, adhesion, and deformation processes. Therefore, the dissipated energy estimated in this study is more appropriately interpreted as an apparent indicator of the evolving contact state rather than as an exact local dissipation quantity. Despite the limited spatial resolution of the LDV measurement relative to the nanoscale contact region, this approach provides cycle-resolved information that reflects the evolution of interfacial behavior during repeated loading.

### 4.3. Limitation and Future Work

A limitation of the current study is the absence of independent replicate experiments using different tips and substrates. Consequently, the reproducibility of the measured wear behavior was not statistically assessed and warrants further investigation in future research.

Although significant insights have been gained, the physical interpretation of the contact states and their associated phase signatures remains incomplete. The spatial resolution of the laser Doppler vibrometry (LDV) signal, which typically measures several micrometers, is considerably larger than the actual contact area, which is quantified in tens of nanometers. Consequently, the LDV response reflects a spatially averaged substrate vibration field rather than the localized dynamics occurring directly within the tip contact zone. High-order or highly localized vibrational components associated with transient contact events can be averaged, leading to the loss of detailed modal information. This averaging effect can influence the measured phase lag and, consequently, affect the estimation of the dissipated energy. Therefore, in this study, the energy estimation should be considered an apparent or effective quantity based on the assumption of viscous force-driven dissipation. Additionally, the signal rectification and processing applied to the LDV output may suppress the fine temporal features of the contact dynamics. Despite these limitations, the current approach demonstrates the potential of LDV-based measurements for characterizing trends in contact behavior. More rigorous contact mechanics modeling and higher-resolution measurements will be necessary in future studies to quantitatively establish accurate energy dissipation mechanisms. Consequently, future research should focus on formulating a multiscale theoretical approach, potentially integrating molecular dynamics with electronic structure calculations, to explicitly correlate the observed contact modes with interfacial bonding states. Furthermore, limitations in observational resolution impede a comprehensive understanding of the proposed interfacial transfer layer. The current SEM and optical observations offer only indirect evidence of material accumulation and transfer at the contact interface, which is inadequate for definitively determining the composition, structure, thickness, or spatial continuity of the interlayer. Consequently, the interpretation of the interlayer-mediated wear behavior in this study should be considered a mechanistic hypothesis supported by the observed tribological trends rather than direct structural verification. Higher-resolution characterization techniques, such as transmission electron microscopy (TEM), are required to resolve the nanoscale morphology and crystallographic structure of the interfacial region. Additionally, advanced chemical and elemental analyses with high spatial resolution, including X-ray photoelectron spectroscopy (XPS) and electron energy loss spectroscopy (EELS), are essential to elucidate the chemical interactions and evolution of the interlayer during repeated sliding.

## 5. Conclusions

The progression of the wear rate and energy dissipation for the Si/Ti, Si/HAp, Ti/Ti, and Ti/HAp material pairs was examined using an AFM system integrated with LDV. The following conclusions were drawn:

- The integration of LDV measurements with velocity–force Lissajous analysis facilitated a cycle-resolved characterization of dynamic contact behavior during repeated sliding. Distinct contact modes were qualitatively discerned from the phase relationship between the substrate vibration velocity and cantilever displacement. Characteristic phase differences were observed to cluster around +π/2, −π/2, and ±π, indicating distinct dynamic responses at the interface. A phase lead approaching π/2 corresponds to synchronized interfacial motion associated with adhesive coupling, whereas phase differences deviating significantly from this condition are indicative of viscoelastic damping-dominated contact behavior. Clarifying the physics of these modes may reveal the nature of the material contact.
- The observed wear rates ranged from approximately $4.46\times 10^{-6}$ to $7.0\times 10^{-3}$ nm/cycle, underscoring the highly localized nature of nanoscale reciprocal wear and the necessity for high-resolution analysis. Negative conventional wear rates observed under several scanning conditions suggest apparent material transfer between the contacting bodies, indicating that diffusion- and transfer-mediated processes significantly contribute to nanoscale wear evolution.
- Heterogeneous cyclic wear is observed at nanoscale contacts. The simultaneous material exchange on both sides of the interface underscores the significant role of diffusion-mediated wear at this scale in the wear process. The fluctuation in the wear rate across the scan cycles confirms the involvement of interlayer formation, which is not captured in conventional wear models.
- The dissipative transfer steady state at nanoscale contacts can be interpreted as a dynamic balance between progressive wear and interlayer-mediated wear. The discrepancies in the energy wear coefficients, approximately two orders of magnitude among the Si/HAp, Si/Ti, and Ti/Ti systems, indicate fundamentally different wear mechanisms governed by both mechanical properties and interfacial interactions.
- Wear mechanisms in complex interfaces such as Ti/HAp challenge conventional energy-based wear models. Variations in the contributions of distinct wear regimes across scan cycles appear to be key to understanding the relationship between energy dissipation and wear behavior in these systems.

A diffusion-mediated material transfer perspective is essential for constructing a comprehensive understanding of wear mechanisms, particularly under localized damage conditions at the nanoscale level. The interlayer, formed as an intrinsic consequence of interfacial diffusion, plays a critical role in governing the wear behavior. Therefore, achieving a predictive wear model requires a deeper insight into the contact dynamics and mechanisms of energy dissipation at the interface.

## Data Availability

The original contributions presented in this study are included in the article. Further inquiries can be directed to the corresponding author.

## Abbreviations

The following abbreviations are used in this manuscript:

Si	Silicon
Ti	Titanium
HAp	Hydroxylapatite
$F_{cant}$	Vertical force by cantilever
$v_{subs}$	Velocity of substrate
γ	Effective damping coefficient
$E_{cycle}$	Energy dissipated in a cycle
k	Effective stiffness of cantilever
$E_{d}$	Representative energy dissipation per cycle
N	Eumber of recorded cycle
$D_{i}$	Average wear depth of scan *i*
Δz	Eean height difference of scan between cycles
$N_{pixel}$	Number of pixel on topology image
$W_{con}$	Conventional wear rate of a scan
$W_{abs}$	Absolute wear rate of a scan
A	Scanned area
$N_{c}$	Number of applied cycle per scan
$r_{c}$	Contact radius
ϕ	Phase difference
LDV	Laser Dopler Vibrometer
SPS	Spark plasma sintering
SPM	Scanning Probe Microscope
SEM	Scanning Electron Microscope
BPF	Band Pass Filter
